# Madecassoside Protects Against LPS-Induced Acute Lung Injury *via* Inhibiting TLR4/NF-κB Activation and Blood-Air Barrier Permeability

**DOI:** 10.3389/fphar.2020.00807

**Published:** 2020-06-05

**Authors:** Lu-Yuan Peng, Hai-Tao Shi, Meng Yuan, Jing-He Li, Ke Song, Jiang-Ni Huang, Peng-Fei Yi, Hai-Qing Shen, Ben-Dong Fu

**Affiliations:** College of Veterinary Medicine, Jilin University, Changchun, China

**Keywords:** Madecassoside, acute lung injury, lipopolysaccharides, alveolar epithelium permeability, Toll-like receptor4/Nuclear factor kappa-B

## Abstract

Madecassoside (MA), a crucial ingredient of *Centella asiatica*, has been reported to exhibit a variety of bioactivities, including antipulmonary fibrosis, and antiinflammatory effects. Here we aimed to elucidate the protective effects and underlying mechanisms of MA on LPS-induced acute lung injury (ALI). The mice were treated with MA for one week and then received intratracheal of LPS to establish the ALI model. Then we evaluated the pathological changes by haematoxylin and eosin staining and measured the levels of proinflammatory cytokines and myeloperoxidase (MPO) by ELISA, the transcriptional level of tight junction proteins by qRT-PCR, as well as the expression of Toll-like receptor4/Nuclear factor kappa-B (TLR4/NF-κB) pathway by Western blot. The results showed that MA significantly inhibited LPS-induced pathological damages, lung edema, MPO, and proinflammatory cytokines production. Furthermore, MA obviously repaired alveolar epithelium integrity showing by reduced secretion of total proteins in the BALF and enhanced mRNA expression of tight junction as Occludin and zonula occludens-1 (ZO-1) comparing to LPS. Further research showed that LPS stimulation activated the TLR4/NF-κB signaling pathway and the activation was inhibited by MA. In conclusion, these data indicated that MA had protective effects against LPS-induced ALI. The therapeutic mechanisms may be associated with reducing the alveolar epithelium permeability and inflammatory response *via* repressing the activation of TLR4/NF-κB pathway.

## Introduction

Acute lung injury (ALI) is a life-threatening clinical syndrome in human. The pathophysiological characteristic of ALI is uncontrolled and acute inflammatory disorder showing by destruction of both capillary endothelial cells and alveolar cells, infiltration of inflammatory mediators into the lung and then inducing the occurrence of ALI caused (Chen et al., 2019; Yang et al., 2019). Lipopolysaccharides (LPS), a main component of the outer membrane of gram-negative, has been applied to be an inducer of ALI (Kristof et al., 1998). In addition, ALI model generated by LPS exposure has been usually applied to mimic and study the features and pathological mechanisms of ALI in humans (Rittirsch et al., 2008). Although a great deal of efforts has been put into finding efficient strategies on ALI, the morbidity and mortality of ALI are still high. Therefore, it is urgently needed to seek for potent drugs that treat ALI by inhibiting the uncontrolled inflammatory responses.

Madecassoside (MA), a major principle terpenoid occurring in *Centella asiatica* herbs, possesses extensive biological activities and has been widely used as an antiapoptosis, antioxidant, and antiinflammatory agent (Cao et al., 2010; Wang et al., 2018). Evidence showed that MA prevented the releases of proinflammatory cytokines by repressing the activation of MAPK and NF-κB signaling pathway and enhancing the expression of heme oxygenase-1 (HO-1) and nuclear factor E2-related factor 2 (Nrf2) during LPS/D-GalN-induced liver injury (Wang et al., 2018). Other reports also demonstrated that MA had antineuroinflammatory effects and concentration-dependently restrained the expression of proinflammatory cytokines, nitric oxide synthase, cyclooxygenase-2, and nuclear factor kappa-B (NF-κB) pathway induced by LPS (Sasmita et al., 2018). Moreover, Cao et al. also proved that MA prevented the LPS-induced sepsis and the protective mechanisms were associated with inhibiting inflammatory response induced by LPS (Cao et al., 2010). However, the effect of MA on ALI caused by LPS is not entirely clear. Therefore, we explored the therapeutic effects and mechanisms of MA on LPS-induced ALI in the present study.

## Materials and Methods

### Animals and Treatment

Adult BALB/c mice (male, 6–8 weeks) were obtained from the Center of Experimental Animals of Changsheng Biotechnology Co. Ltd (Liaoning, China) and kept in standard cages with adequate food and water. The temperature and humidity of room were controlled. MA was purchased from Chengdu Must Biotechnology Co., Ltd. (Chengdu, China).

Total 48 mice were randomly separated into six groups (n=8), including control group, MA alone group (40 mg/kg), LPS group, MA (10, 20, and 40 mg/kg) + LPS group. ALI model was established by intranasal perfusion of 5 mg/kg of LPS dissolved into sterile saline as described (Ye and Liu, 2019). Mice in MA + LPS groups were orally administered for one week (one hour before LPS injection in the last day) of 10, 20, and 40 mg/kg MA at a volume of 200 μl. Twenty-four hours later, animals were euthanized and the biological samples were collected for further detection. All experiments complied with the manual of the care and use of laboratory animals published by the Institutional Animal Care and Use Committee of Jilin University for animal experiments approvals published by Jilin University (Number of permit: KT201903080).

### Pathological Assay

The partial sections of lung tissues were removed and fixed with 4% paraformaldehyde for 48 h, then dehydrated, and embedded in paraffin. Paraffin-embedded tissues were cut into 5-μm thick pieces, and then dehydrated, stained by hematoxylin and eosin (H&E). The pathological damages were examined using a light microscopy. In addition, lung tissue injury was scored by two pathologists blind to group assignment according to previous described (Hu et al., 2019). According to the degree of alveolar hyperemia, hemorrhage, infiltration or aggregation of neutrophils in the alveolar space or vascular wall, and thickening of the alveolar walls and/or formation of hyaline membranes, the lung injury was scored from 0 to 4 (0: no obvious lesions; 1: mild lesions; 2: moderate lesions; 3: severe lesions; and 4: obvious severe lesions).

### MPO Measurement

The lung tissue was weighed and homogenized with phosphate buffer solution (PBS) to obtain 10% homogenate. Then, the homogenate was centrifuged and the supernatant was removed to measure the production of MPO *via* an ELISA kit according to the manufacturer’s instructions (Lengton Bioscience Company, Shanghai, China), normalized to total protein as assessed by a BCA kit (Thermo, MA, USA) as previously described (Maia et al., 2019).

### Lung Wet/Dry Ratio Assay

The degree of pulmonary edema was determined by the wet weight to dry weight. Briefly, the fresh lung tissues from all groups were removed and cut to obtain wet weight. Later, the samples were desiccated in an oven at 80°C for 48 h to obtain the dry weight. The values of wet weight dividing to dry weight were considered as the ratio of wet to dry.

### Bronchoalveolar Lavage Fluid Collection and Treatment

Twenty-four hours after LPS infusion, the mice from each group were sacrificed and exposed the trachea. A sterile 18 G trocar inserted into the trachea and thread with silk. The left lung was lavaged with sterile and ice of PBS (0.4 ml each time) for three times. The bronchoalveolar lavage fluid (BALF) samples were centrifuged at 2,000*g* for 10 min at 4°C. The supernatant was collected and stored at −80°C for subsequent detection. The supernatant was used to measure the concentration of total protein in BALF by BCA kits (Thermo, MA, USA). The precipitated cells were suspended with ice PBS, smeared and stained with Diff-Quik stain to count the number of total cells by a cell counting plate under optical microscope.

### ELISA Assay

After LPS challenge for 24 h, the BALF was collected for the measurement of TNF-α and IL-1β. And the right upper lung was immediately frozen and homogenized in ice-cold PBS by a tissue grinder (Servicebio KZ-II, Wuhan, China) at 4°C for 10 min to obtain 10% homogenate. After centrifuged at 2,000*g* for 10 min at 4°C, the concentration of TNF-α and IL-1β in supernatant were measured by ELISA kits following the manufacturer’s procedures (Biolegend, San Diego, USA), normalized to total protein as assessed by a BCA kit (Thermo, MA, USA) as previously described (Maia et al., 2019).

### Real-Time Quantitative PCR

0.1-g lung tissues of each group was weighed and dissociated with 1-ml TRIzol regent. Then, the concentration of extracted RNA was estimated using a Q6000 system (Quawell Technology, USA). After the RNA was reversed to cDNA, the mRNA expression of tight junction proteins including ZO-1, Occludin was tested through the 7,500 real-time PCR system (Applied Biosystems, Foster, CA). The primers referenced in this study were listed in **Table 1**.

**Table 1:** Primers for real-time quantitative PCR (qRT-PCR).

Primer name	Sequence (5′ to 3′)
*ZO-1-F*	GACCTTGATTTGCATGACGA
*ZO-1-R*	AGGACCGTGTAATGGCAGAC
*Occludin-F*	ACACTTGCTTGGGACAGAGG
*Occludin-R*	AAGGAAGCGATGAAGCAGAA
*β-actin -F*	GTCAGGTCATCACTATCGGCAAT
*β-actin -R*	AGAGGTCTTTACGGATGTCAACGT

### Western Blot

The tissues were cut, lysed by a protein extract reagent kit (Thermo, MA, USA), and measured the concentration of total proteins through a BCA kit (Thermo, MA, USA). The total proteins were separated by 12% SDS-PAGE and transferred onto PVDF membranes. After blocking with 5% skimmed milk, the membranes were incubated with the primary antibodies including TLR4, NF-κB p65, NF-κB pp65, NF-κB IκBα, NF-κB pIκBα (Biosynthesis Biotechnology Inc. Beijing, China) for a night. Finally, the cleaned membranes were followed by 1.5h incubation with secondary antibody (Biosynthesis Biotechnology Inc. Beijing, China) and detected using an enhanced chemiluminescence (ECL) Western blotting detection reagents (Merck, Massachusetts, USA).

### Statistical Analysis

All experimental data were expressed as the means ± S.D. Statistical analysis was carried out in one-way analysis of variance (ANOVA) by Tukey-Kramer multiple comparison using GraphPad Prism 6.0. *p* < 0.05 was considered statistically significant.

## Results

### MA Alleviated Lung Pathological Injury Induced by LPS

**Figure 1:**
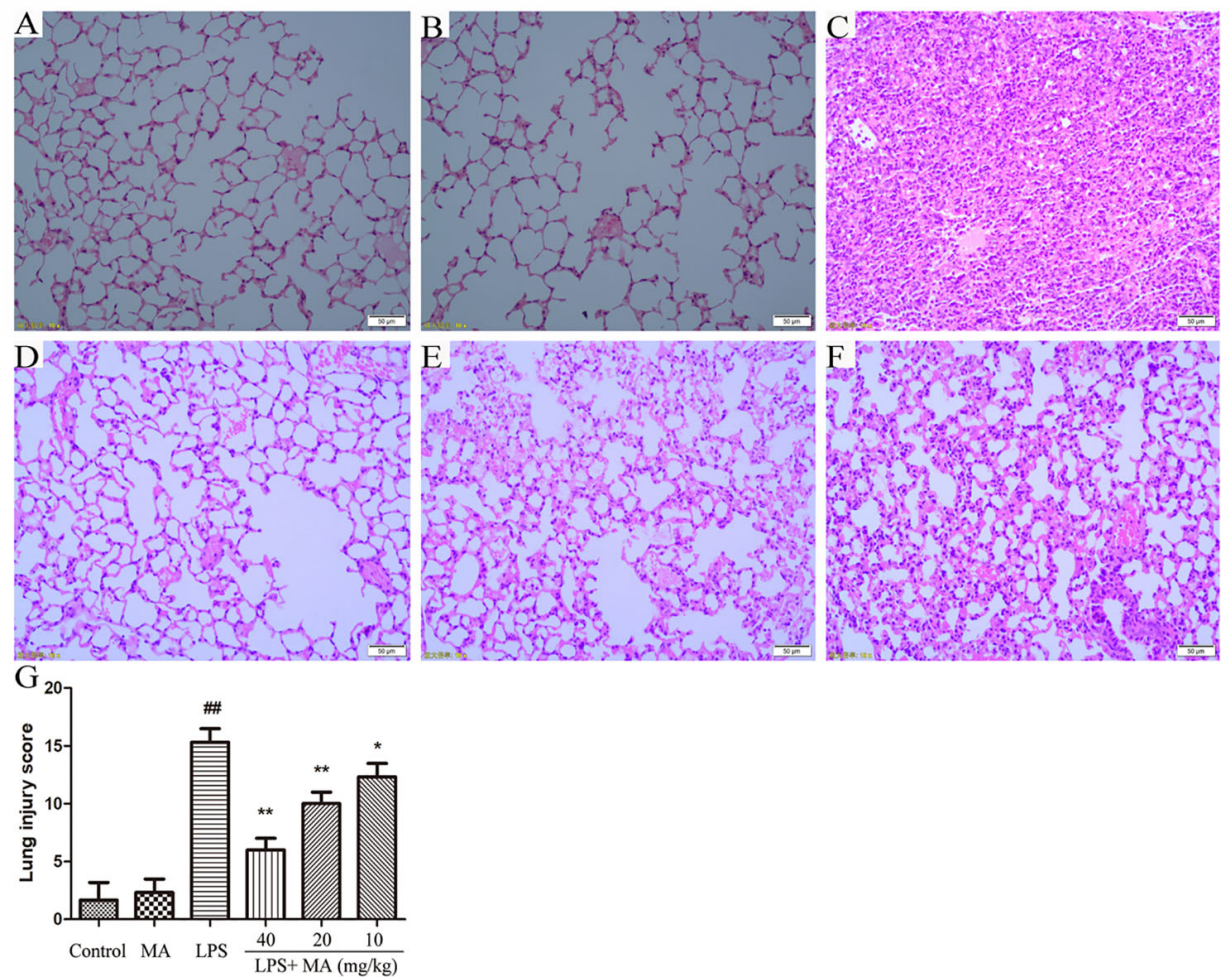
Pathohistological evaluations of lung tissues and lung injury scoring. Hematoxylin and eosin (H&E) staining of lung tissues in **(A)** control, **(B)** Madecassoside (MA) alone, **(C)** Lipopolysaccharides (LPS) and **(D–F)** LPS+MA (40, 20, 10 mg/kg) groups. **(G)** Lung injury score of all groups. Mean ± S.D. (n=8). ^##^ *p* < 0.01: significantly different from control; ^*^ *p* < 0.05 and ^**^ *p* < 0.01: significantly different from the LPS group.

As shown in **Figures 1A, B**, the mice in the control group and MA alone group showed normal architecture morphologically while the mice in LPS group showed significantly lung pathological damage including hemorrhage in the alveolar spaces, thickened alveolar walls, and inflammatory cells infiltration (**Figure 1C**). However, these changes were obviously alleviated by MA in a dose dependent manner (**Figures 1D–F**). In addition, lung injury score also indicated that MA significantly inhibited the lung pathological damages induced by LPS (**Figure 1G**).

### MA Reduced Lung MPO Activity Induced by LPS

**Figure 2:**
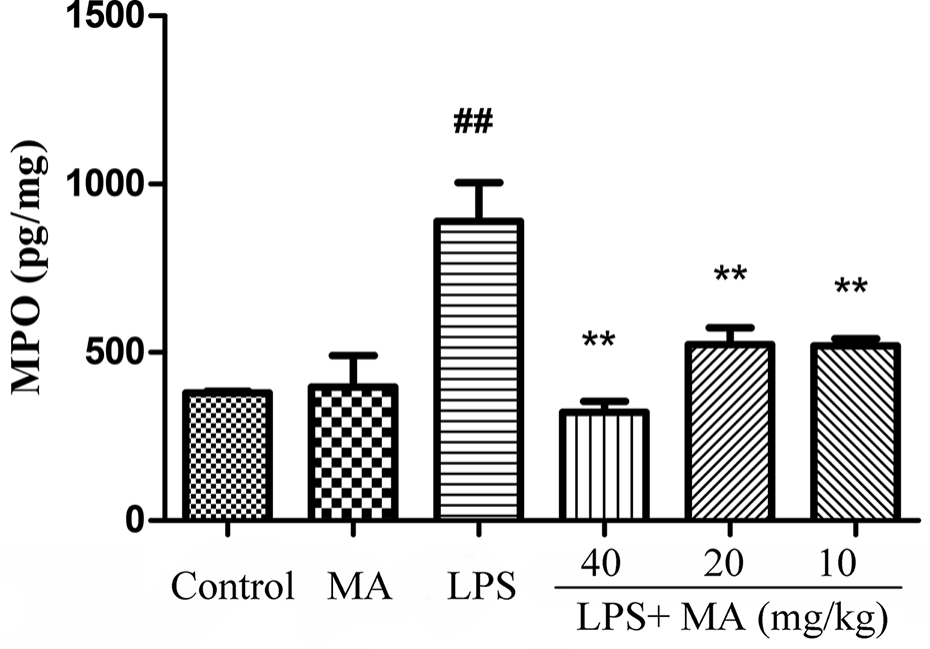
Myeloperoxidase (MPO) assay of lung injury was measured by ELISA. Mean ± S.D. (n=8). ^##^ *p*<0.01: significantly different from control; ^**^ *p* < 0.01: significantly different from the lipopolysaccharides (LPS) group.

Lung MPO was regarded as an index to evaluate the pulmonary inflammatory damage. As **Figure 2** revealed, the releases of MPO were markedly increased from the LPS group in comparison with the control group. However, MA treatment significantly reduced the release of MPO induced by LPS. In addition, MPO secretion in MA alone group had no difference from control group.

### MA Ameliorated Lung Edema Induced by LPS

**Figure 3:**
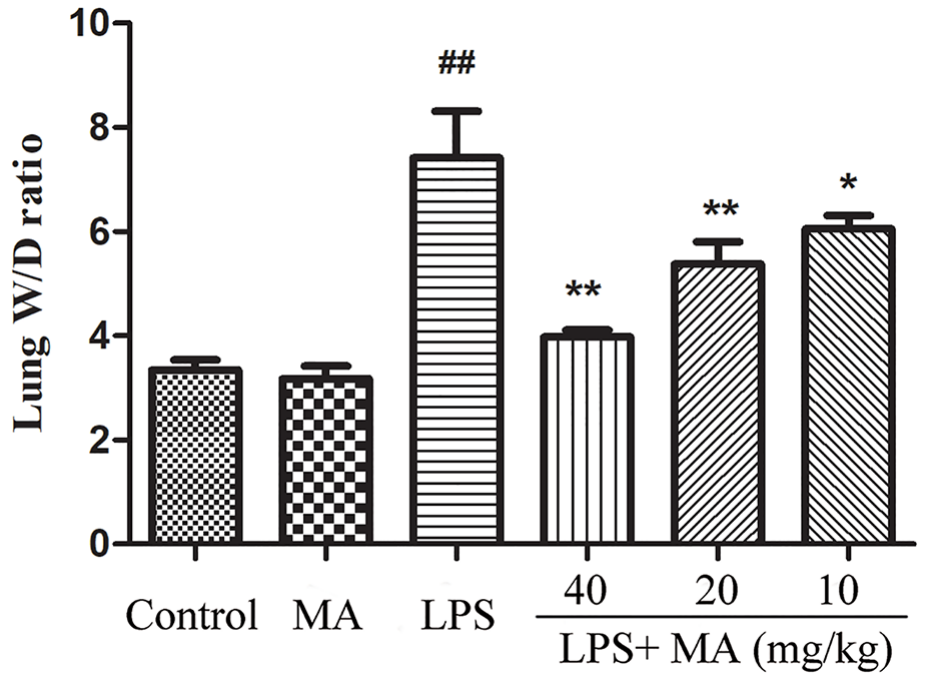
Wet weight to dry weight ratio of lung tissues. Mean ± S.D. (n=8). ^##^ *p* < 0.01: significantly different from control; ^*^ *p* < 0.05 and ^**^ *p* < 0.01: significantly different from the lipopolysaccharides (LPS) group.

The wet/dry ratio of ALI mice was measure to assess the severity of pulmonary edema. In the **Figure 3**, the results showed that lung wet/dry ratio was remarkably elevated after LPS stimulation. Conversely, the increased lung wet/dry ratio induced by LPS was distinctly attenuated by MA during ALI stage. MA alone had no effect on wet/dry ratio.

### MA Treatment Maintained the Integrity of Air-Blood Barrier

**Figure 4:**
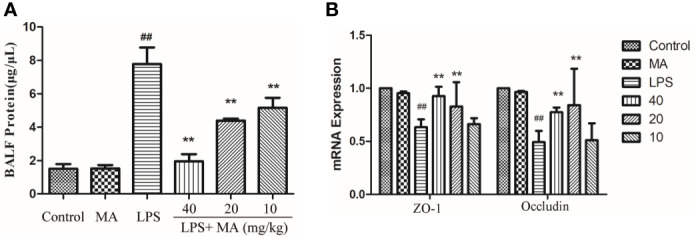
Air-blood barrier permeability examination. **(A)** Total protein in bronchoalveolar lavage fluid (BALF) and **(B)** mRNA expression of ZO-1 and Occludin in lung tissues were detected to access the integrity of air-blood barrier. Mean ± S.D. (n=8). ^##^ *p* < 0.01: significantly different from control; ^**^ *p* < 0.01: significantly different from the lipopolysaccharides (LPS) group.

The concentration of total protein in BALF was usually applied to assess the integrity of air-blood barrier. As the **Figure 4A** showed, MA treatment dose-dependently suppressed the upregulation of total proteins in the BALF during ALI induced by LPS. Moreover, maintaining the air-blood barrier integrity required the involvement of tight junction proteins such as Occludin and ZO-1. Our data in this study showed that the mRNA expression of Occludin and ZO-1 was significantly downregulated by LPS. However, the reduced expression of tight junction induced by LPS were clearly reversed by MA treatment. Oral administration of MA alone had no effect on the indicators of air-blood barrier (**Figure 4B**).

### MA Alleviated Inflammatory Responses in the Lung During ALI

**Figure 5:**
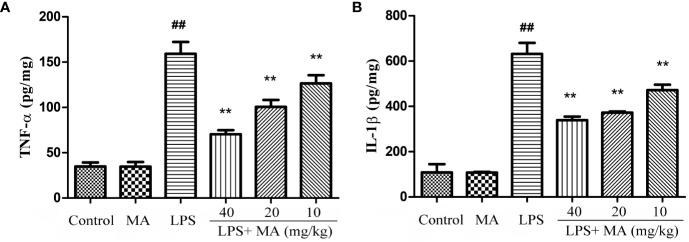
The measurement of TNF-α and IL-1β in lung tissues. Lung tissue was homogenized in PBS and the concentrations of **(A)** TNF-α and **(B)** IL-1β (pg per mg protein) measured in the supernatant. Mean ± S.D. (n=8). ^##^ *p* < 0.01: significantly different from control; ^**^ *p* < 0.01: significantly different from the lipopolysaccharides (LPS) group.

The production of inflammatory cytokines was considered as an index to evaluate the degree of inflammation. In our study, the effect on the concentration of TNF-α and IL-1β was tested to assess the antiinflammatory activity of MA. Data in **Figure 5** showed that LPS challenge significantly increased the production of TNF-α and IL-1β. However, MA dose-dependently restrained the increase of these proinflammatory cytokines during the ALI stage. MA alone showed no difference in the production of TNF-α and IL-1β from control group.

### MA Reduced Cell Infiltration Induced by LPS

**Figure 6:**
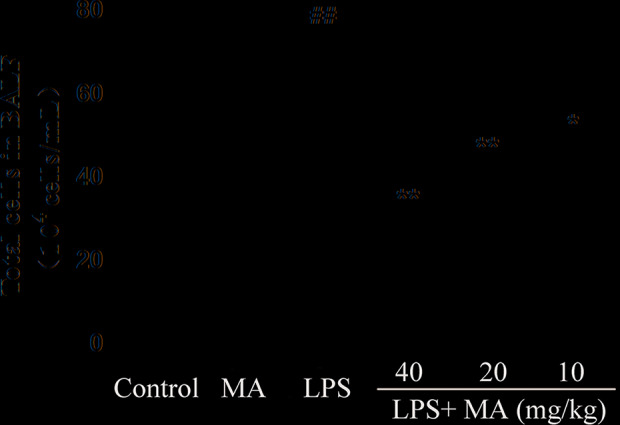
Inflammatory cell infiltration in bronchoalveolar lavage fluid (BALF). Mean ± S.D. (n=8). ^##^ *p* < 0.01: significantly different from control; ^*^ *p* < 0.05 and ^**^ *p* < 0.01: significantly different from the lipopolysaccharides (LPS) group.

The results showed that oral administration of MA alone had no influence on the releases of TNF-α and IL-1β, while LPS challenged led to marked inflammatory cells infiltration in the BALF. However, MA dose-dependently reduced the count of total cells in the BALF (**Figure 6**).

### MA Reduces Cytokines Production in the BALF Induced by LPS

**Figure 7:**
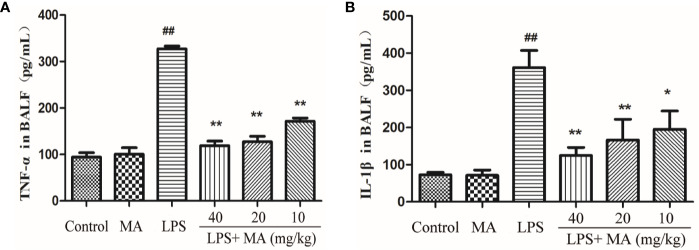
TNF-α and IL-1ß concentrations in bronchoalveolar lavage fluid (BALF). The secretion of **(A)** TNF-α and **(B)** IL-1β were measured by ELISA kits. Mean ± S.D. (n=8). ^##^ *p* < 0.01: significantly different from control; ^*^ *p* < 0.05 and ^**^ *p* < 0.01: significantly different from the lipopolysaccharides (LPS) group.

The secretion of TNT-α and IL-1β in BALF was also detected to evaluate the degree of lung inflammation. As shown in **Figure 7**, MA dose dependently suppressed the production of TNF-α and IL-1β in the BALF during LPS-induced lung damage. Oral administration of MA alone had no effect on the production of TNF-α and IL-1β comparing to control group.

### MA Inhibited TLR4/NF-κB Signaling Pathway Activation in the Lung Induced by LPS

**Figure 8:**
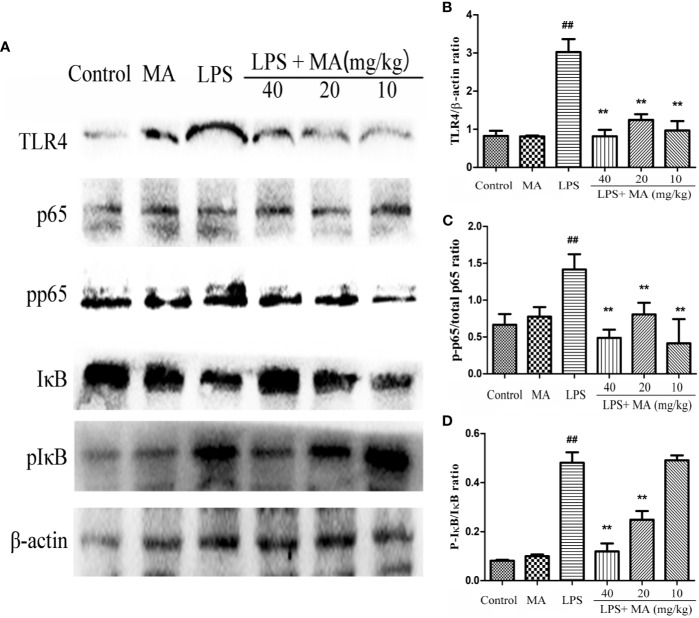
The activation of TLR4/NF-κB pathway. **(A)** TLR4/NF-κB protein samples were analyzed by Western blot with TLR4, IκB, p-IκB, p65, p-p65 antibodies. β-actin was used as a control. **(B)** Quantification of TLR4 proteins and **(C)** p-p65, **(D)** p-IκB protein was determined by densitometry. Mean ± S.D. (n=8). ^##^ *p* < 0.01: significantly different from control; ^**^ *p* < 0.01: significantly different from the lipopolysaccharides (LPS) group.

To explore the antiinflammatory mechanism of MA on LPS-induced ALI, we detected the activation of TLR4/NF-κB signaling pathway in the present study. As shown in **Figure 8**, the expression of TLR4, phosphorylation of NF-κB p65, and phosphorylation of IκBα were significantly increased in the LPS group compared with the control group. However, the increased expression of TLR4, phosphorylation of NF-κB p65 and phosphorylation of IκBα was inhibited by the MA treatment when compared to the LPS group. In addition, MA alone had no influence on TLR4/NF-κB signaling pathway activation. The original images of western blot were shown in **Supplementary Material**.

## Discussion

ALI is a complex clinical condition characterized by severe pulmonary inflammatory responses, parenchymal damages and lung epithelium disruption (Togbe et al., 2007; Chen et al., 2010). Antiinflammatory treatments represent the main therapeutic approaches against ALI. Therefore, it is currently vital to seek effective antiinflammatory medicine for the treatment of ALI. Previous studies have reported that MA ameliorates bleomycin-induced pulmonary fibrosis in mice (Lu et al., 2014; Xia et al., 2016) and protected mice against doxorubicin-induced nephrotoxicity (Su et al., 2015). In the current study, we explored the protective effects and mechanisms of MA on ALI mice model. The results indicated that MA had ability to alleviate LPS-induced ALI through reducing pathological damages, MPO production, pulmonary edema, proinflammatory cytokines production, and enhanced tight junction proteins expression. Additionally, MA also lightened the levels of total proteins, cell infiltration, and production of TNF-α, IL-1β in the BALF during ALI stage. In further study, we found that the antiinflammatory mechanisms of MA on LPS-induced ALI may be through inhibiting the activation of TLR4/NF-κB signaling pathway.

At the stage of ALI, there are a large amounts of protein-rich edema fluid infiltrated into the alveoli and interstitium due to the damaged integrity of alveolar-capillary membrane and impaired alveolar fluid clearance capability of the alveolar epithelium (Herrero et al., 2019). Our study suggested that MA treatment significantly restricted the LPS-induced pulmonary edema because of the lowered lung wet/dry ratio of mice from MA treatment group comparing to that of the LPS group. The levels of total proteins in the BALF were served as an indicator of the permeability of epithelial barrier. Pretreatment with different doses of MA also significantly reduced the production of total proteins in the BALF induced by LPS. In addition, the tight junctions that present in alveolar epithelium, including Occludin and ZO-1, play an important role in maintaining the permeability of alveolar epithelium. They are intercellular structures that prevent the passage of molecules from crossing the paracellular spaces (Herrero et al., 2019). In the present study, we found that the mRNA expression of Occludin and ZO-1 were significantly downregulated in the mice from LPS group compared to the control group. However, MA treatment obviously upregulated the expression of Occludin and ZO-1 in the lung tissues. These results suggested that the protective effects of MA on LPS-induced ALI may be associated with the ability of maintaining the alveolar epithelium integrity.

The major feature of ALI is uncontrolled inflammatory responses in the lung. Proinflammatory cytokines, such as TNF-α and IL-1β, were regarded as one of the most important indicators of inflammation. TNF-α and IL-1β both participated in the inflammation in the early stage, and they were associated with activating and recruiting neutrophils to the site of infection (Miao et al., 2007). In the present study, we found that TNF-α and IL-1β were significantly increased in both the lung tissues and the BALF from the LPS group. However, the increased cytokines were obviously inhibited by MA. MPO is mainly synthesized and expressed in neutrophils and MPO activity often serves as an indicator of activation and accumulation of neutrophils in the site of infection (Fu et al., 2019; Qin et al., 2019). In addition, cell infiltration in the lung tissues is also recognized as one of the most important factors in the development of ALI. The present study showed that different concentrations of MA obviously inhibited the MPO production in LPS-challenged mice. These results suggested that MA had antiinflammatory effects on LPS-induced ALI.

NF-κB pathway is linked to the cytokines production and plays a pivotal role in regulating the inflammatory responses (Markopoulos et al., 2018; Fu et al., 2019). Some studies have indicated that excitation of NF-κB signaling pathway was closely connected with the development of ALI. Targeting to the regulation of NF-κB pathway can effectively control the occurrence of ALI (Jin et al., 2018; Meng et al., 2018; Tian et al., 2019). In unstimulated station, NF-κB p65 is binded to IκBα and exists in inactivation state. After LPS stimulation, the IκB protein is degraded and the NF-κB p65 is translocated into the nucleus from cytoplasm, initiating the transcription of proinflammatory cytokines and inducing inflammation (Xia et al., 2009; Li et al., 2018; Fu et al., 2019). The present study suggested that different concentrations of MA obviously inhibited the activation of NF-κB signaling pathway induced by LPS. In addition, TLR4 is identified as a mammalian signaling receptor for LPS, and also participates in the development of inflammatory response (Akira et al., 2001; Tan et al., 2018). Thus, the effect of MA on the activation of TLR4 in the lung tissues was also detected in the present study. The results showed that LPS significantly upregulated the expression of TLR4. However, this change was reversed by MA. These results suggested that antiinflammatory mechanisms of MA may be through inhibiting the activation of TLR4/NF-κB signaling pathway induced by LPS.

Taken together, our study provided the evidences that pretreatment of MA contributed to the protection against LPS-induced ALI. The potential mechanisms may relate to enhance the integrity of air-blood barrier and inhibited the inflammatory responses *via* restraining the activation of TLR4/NF-κB signaling pathway.

## Data Availability Statement

All datasets generated for this study are included in the article/**Supplementary Material**.

## Ethics Statement

The animal study was reviewed and approved by Institutional Animal Care and Use Committee of Jilin University for animal experiments approvals published by Jilin University.

## Author Contributions

B-DF, L-YP, H-QS conceived the idea for this study. MY and H-TS interpreted the data and drafted the figures. J-HL, J-NH and KS performed the statistical tests. L-YP conducted the study and wrote the original draft. All authors listed have made substantial, direct, and intellectual contribution to the work and approved it for publication.

## Funding

This work was supported by the National Natural Science Foundation of China (no. 31972724 and no. 31372470) and the Special Fund for Agro-scientific Research in the Public Interest (201403051).

## Conflict of Interest

The authors declare that the research was conducted in the absence of any commercial or financial relationships that could be construed as a potential conflict of interest.

## Supplementary Material

Data Sheet 1 The original images of western blot. The strips from left to right are control group, MA alone group, LPS group and LPS+MA (40, 20, 10 mg/kg).
