# Supplementary figures and images for: Madecassoside Protects Against LPS-Induced Acute Lung Injury via Inhibiting TLR4/NF-κB Activation and Blood-Air Barrier Permeability
Source: Front Pharmacol. 2020 Jun 5;11:807. doi: 10.3389/fphar.2020.00807 (PMC7289980; doi:10.3389/fphar.2020.00807)

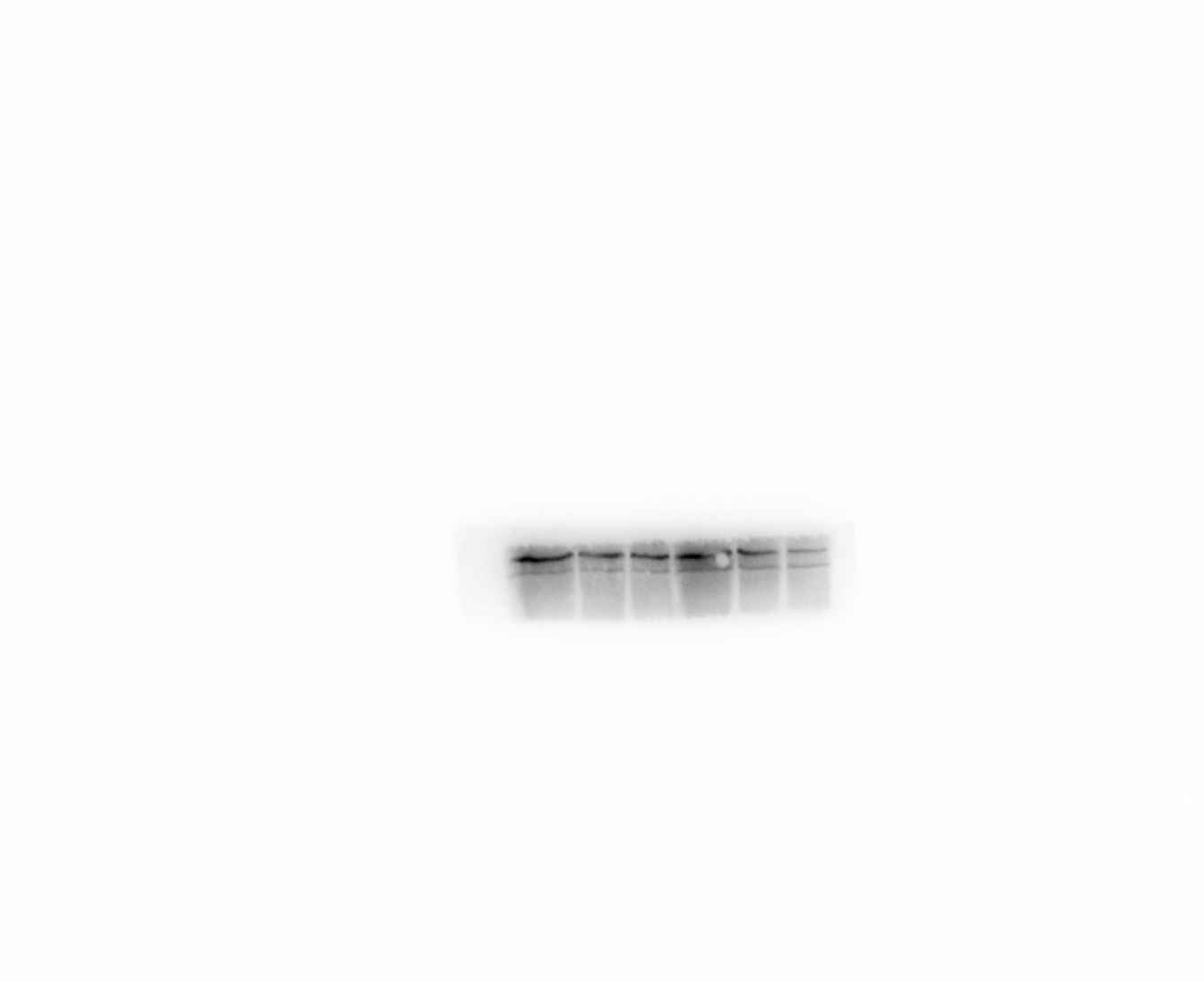

Supplement: Data Sheet 1 — The original images of western blot. The strips from left to right are control group, MA alone group, LPS group and LPS+MA (40, 20, 10 mg/kg). [file DataSheet_1.zip › Original image/IKB/IKB-1.Tif]

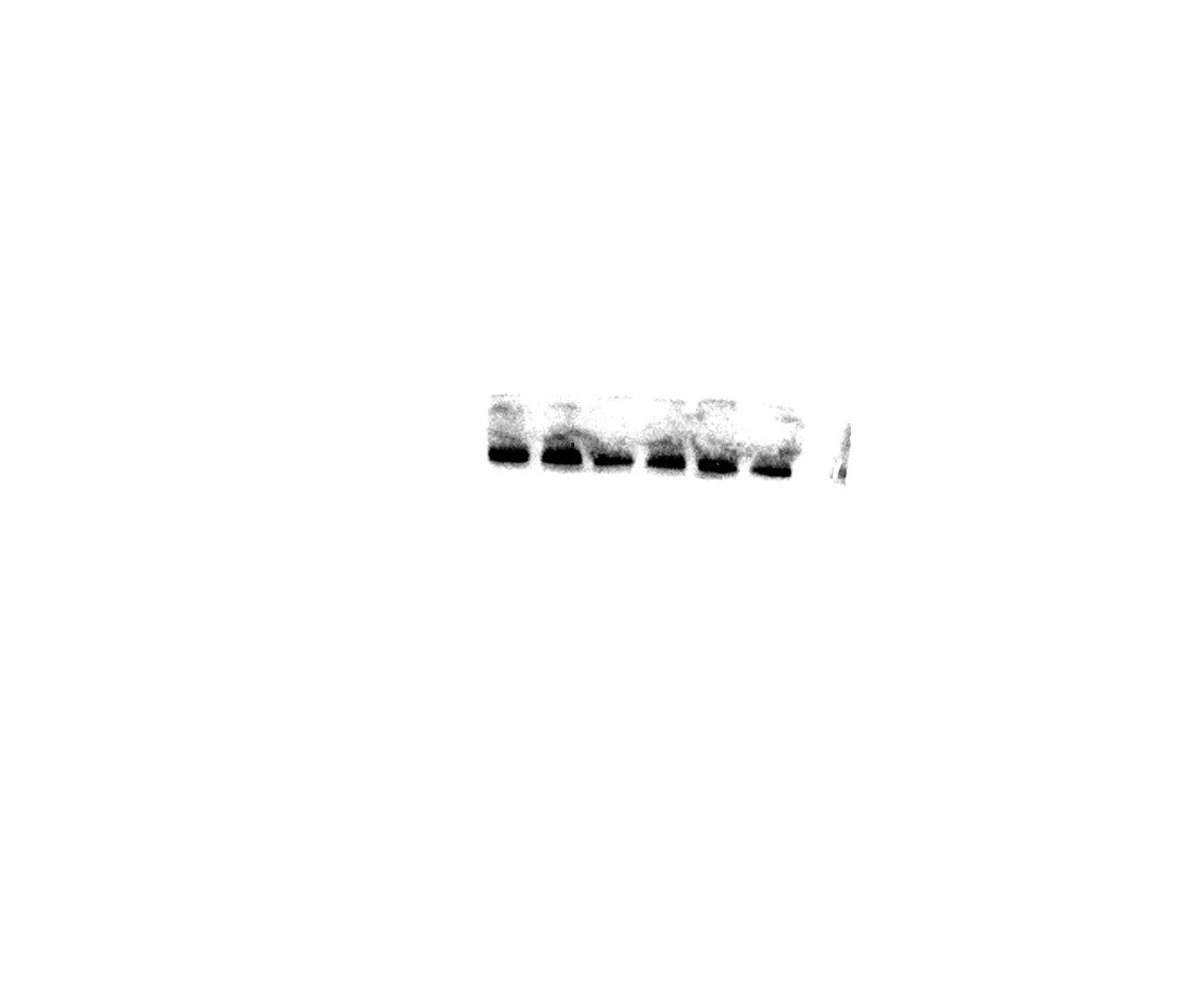

Supplement: Data Sheet 1 — The original images of western blot. The strips from left to right are control group, MA alone group, LPS group and LPS+MA (40, 20, 10 mg/kg). [file DataSheet_1.zip › Original image/IKB/IKB-2.Tif]

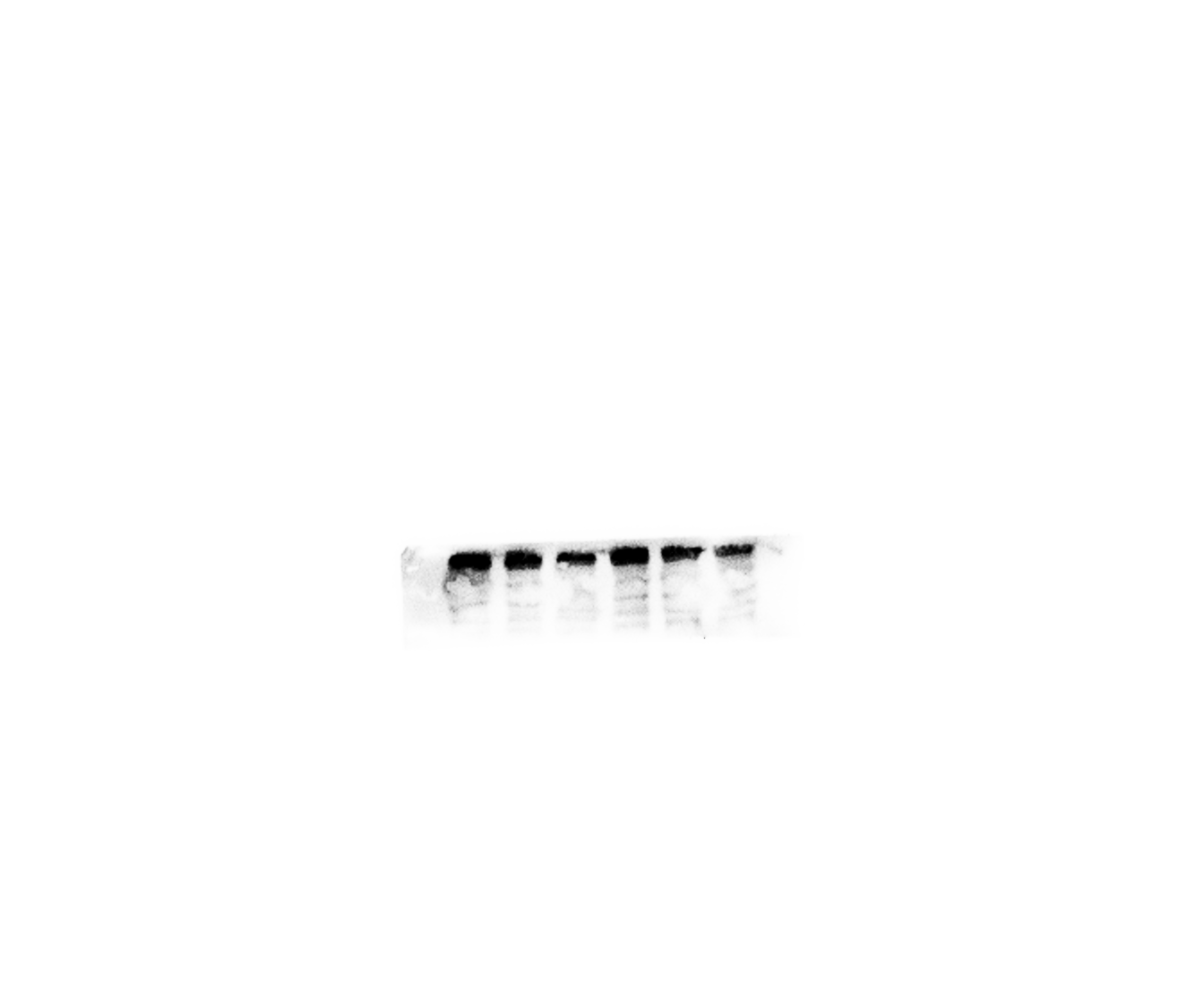

Supplement: Data Sheet 1 — The original images of western blot. The strips from left to right are control group, MA alone group, LPS group and LPS+MA (40, 20, 10 mg/kg). [file DataSheet_1.zip › Original image/IKB/IKB-3.tif]

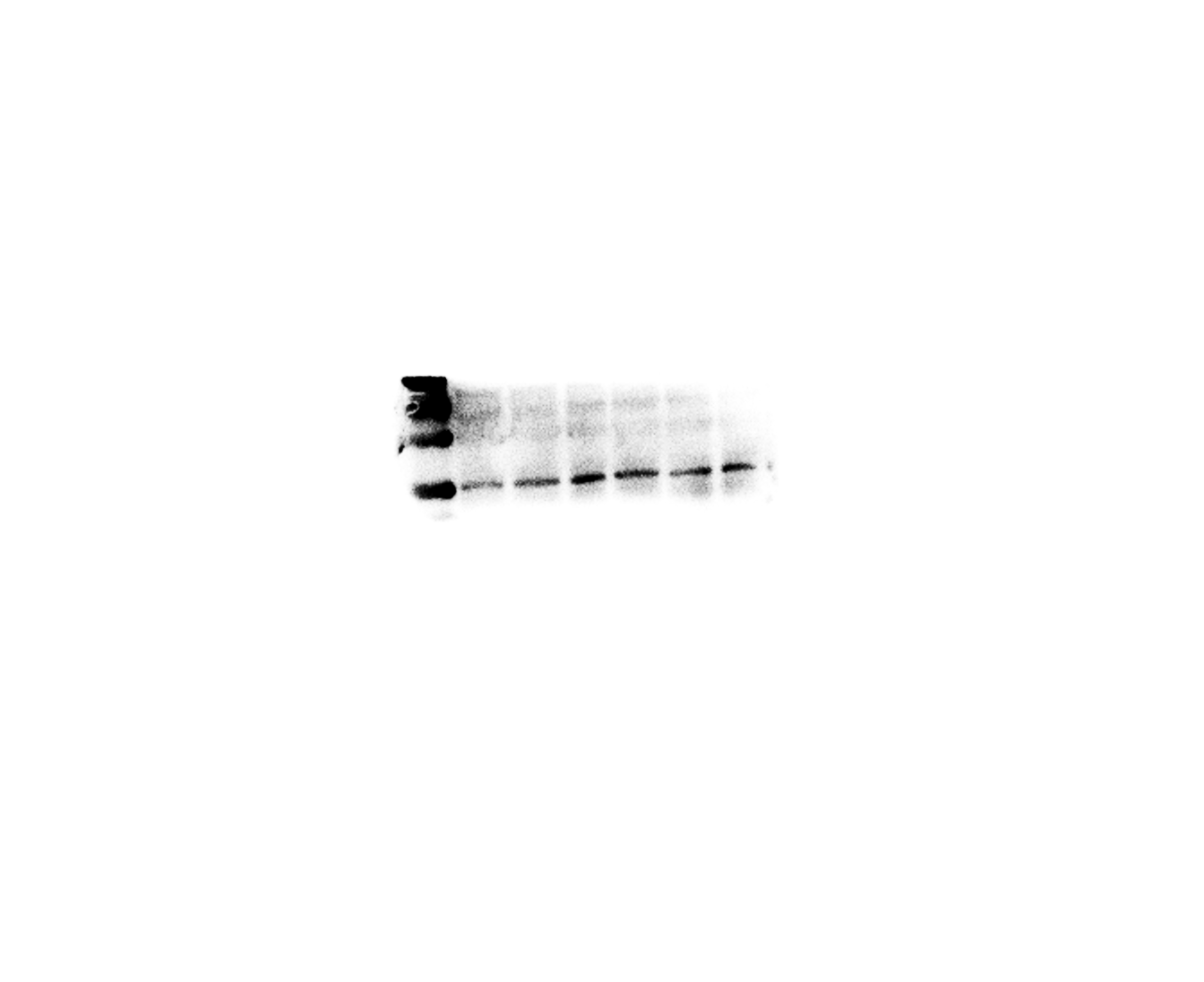

Supplement: Data Sheet 1 — The original images of western blot. The strips from left to right are control group, MA alone group, LPS group and LPS+MA (40, 20, 10 mg/kg). [file DataSheet_1.zip › Original image/TLR4/TLR4-1-1.tif]

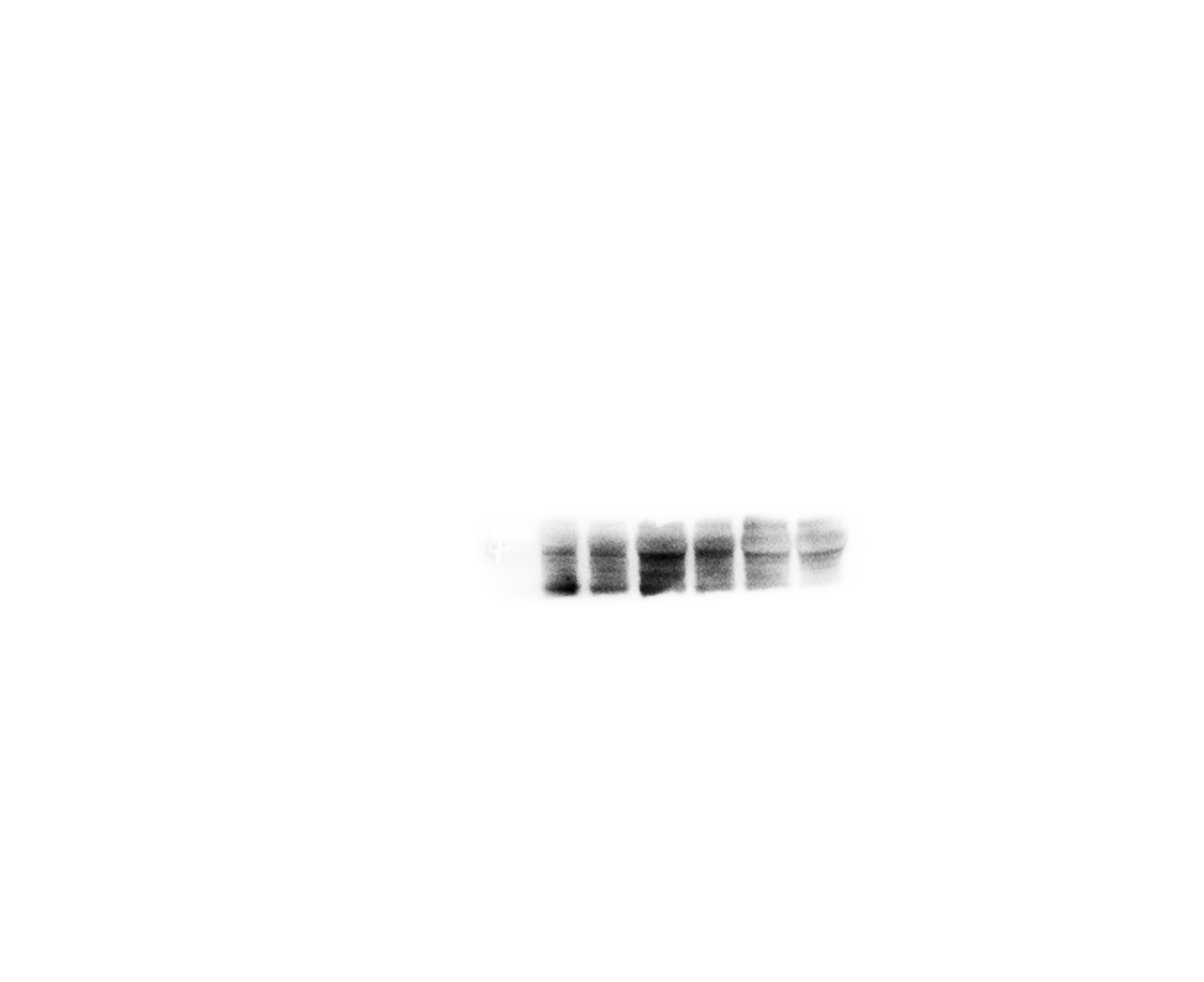

Supplement: Data Sheet 1 — The original images of western blot. The strips from left to right are control group, MA alone group, LPS group and LPS+MA (40, 20, 10 mg/kg). [file DataSheet_1.zip › Original image/TLR4/TLR4-2.tif]

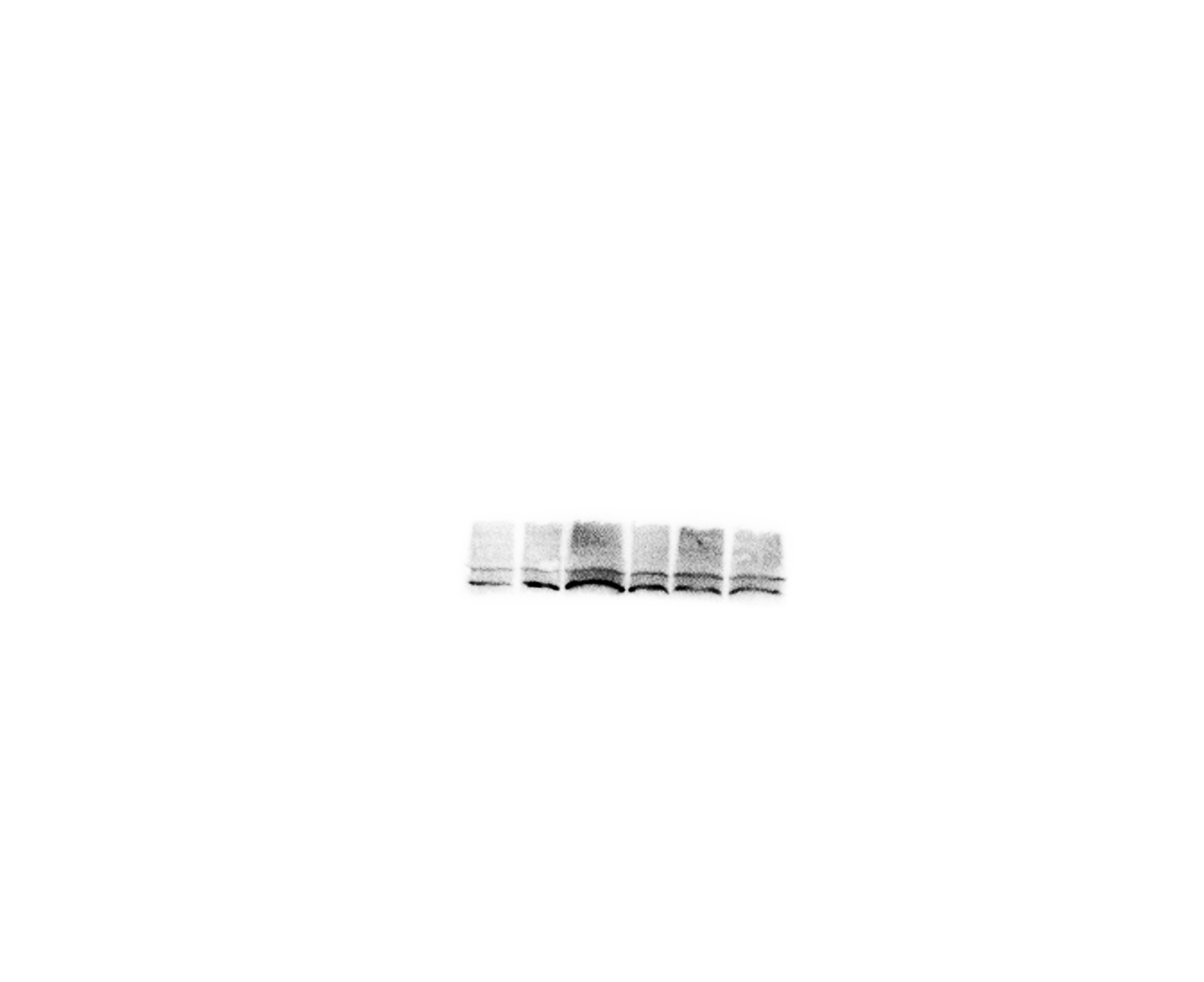

Supplement: Data Sheet 1 — The original images of western blot. The strips from left to right are control group, MA alone group, LPS group and LPS+MA (40, 20, 10 mg/kg). [file DataSheet_1.zip › Original image/TLR4/TLR4-3.tif]

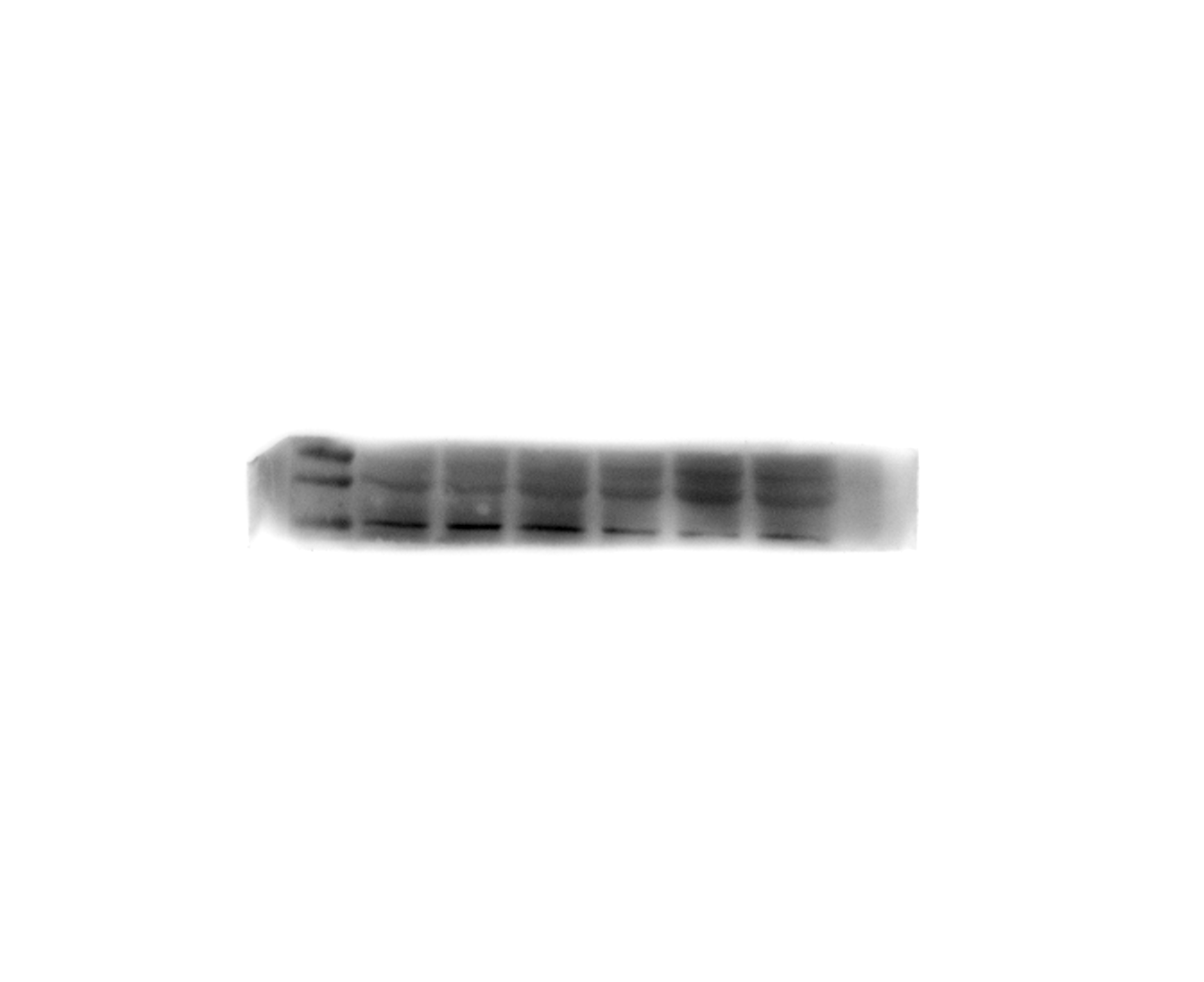

Supplement: Data Sheet 1 — The original images of western blot. The strips from left to right are control group, MA alone group, LPS group and LPS+MA (40, 20, 10 mg/kg). [file DataSheet_1.zip › Original image/actin/actin-1.Tif]

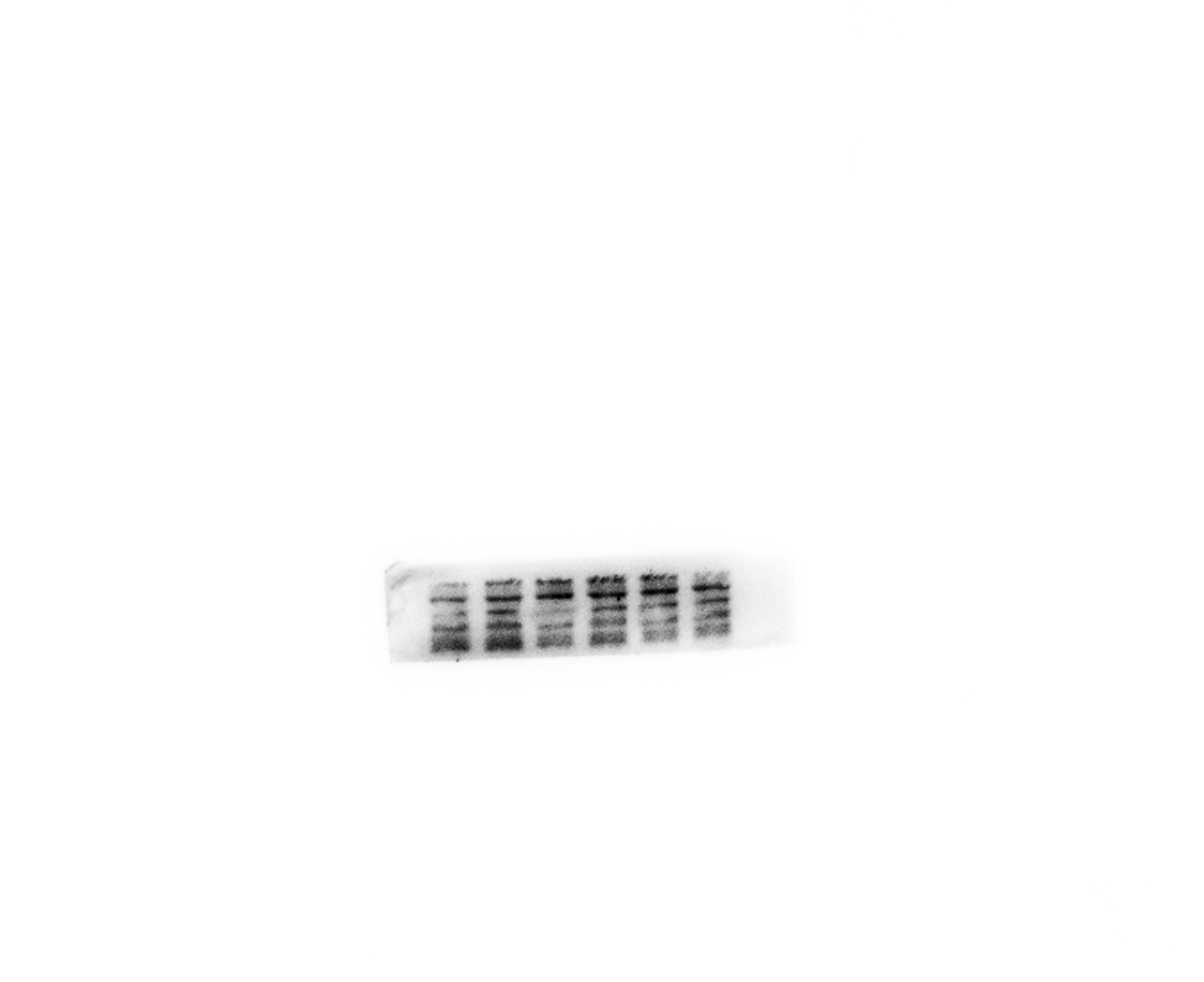

Supplement: Data Sheet 1 — The original images of western blot. The strips from left to right are control group, MA alone group, LPS group and LPS+MA (40, 20, 10 mg/kg). [file DataSheet_1.zip › Original image/actin/actin-2.tif]

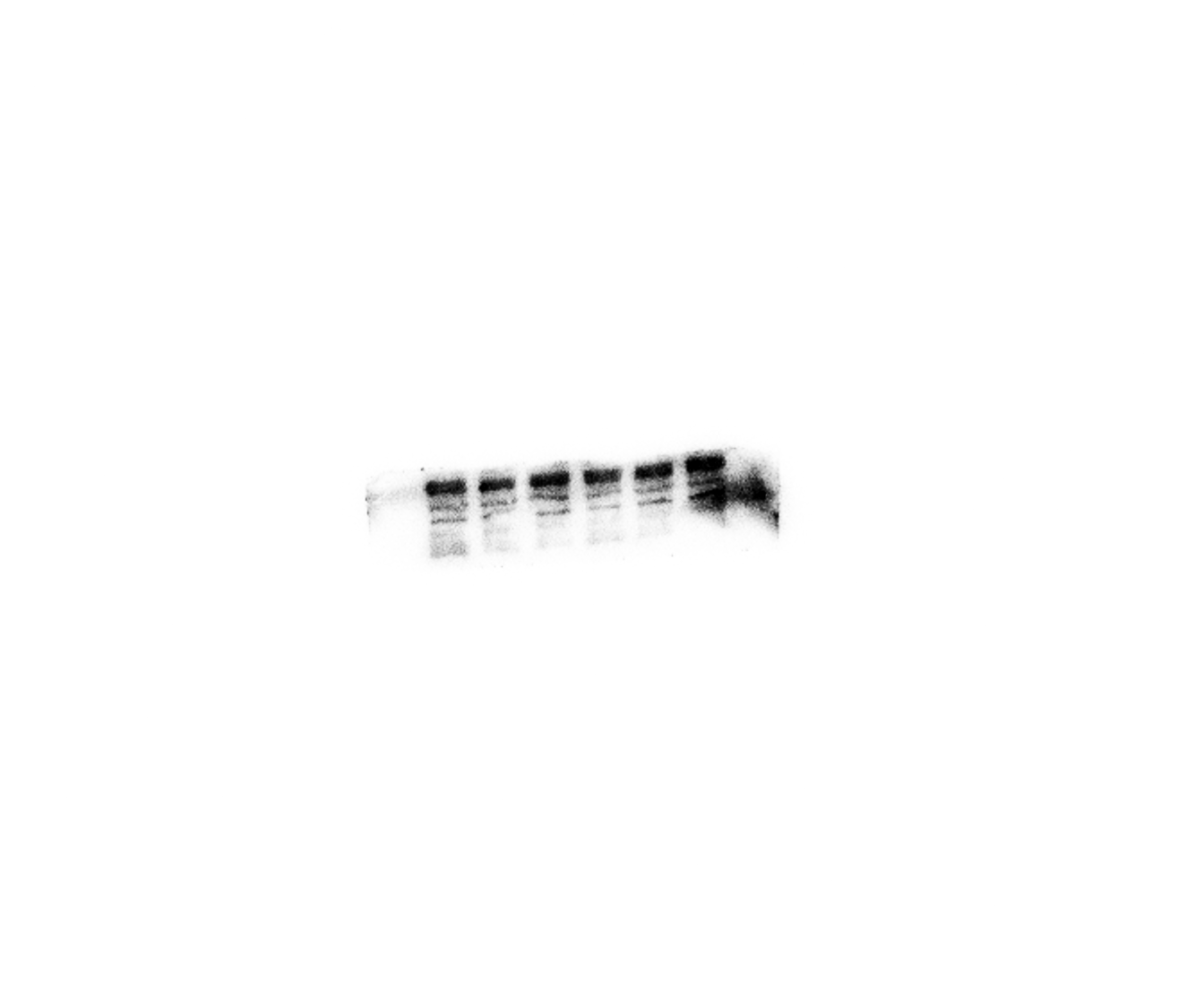

Supplement: Data Sheet 1 — The original images of western blot. The strips from left to right are control group, MA alone group, LPS group and LPS+MA (40, 20, 10 mg/kg). [file DataSheet_1.zip › Original image/actin/actin-3.tif]

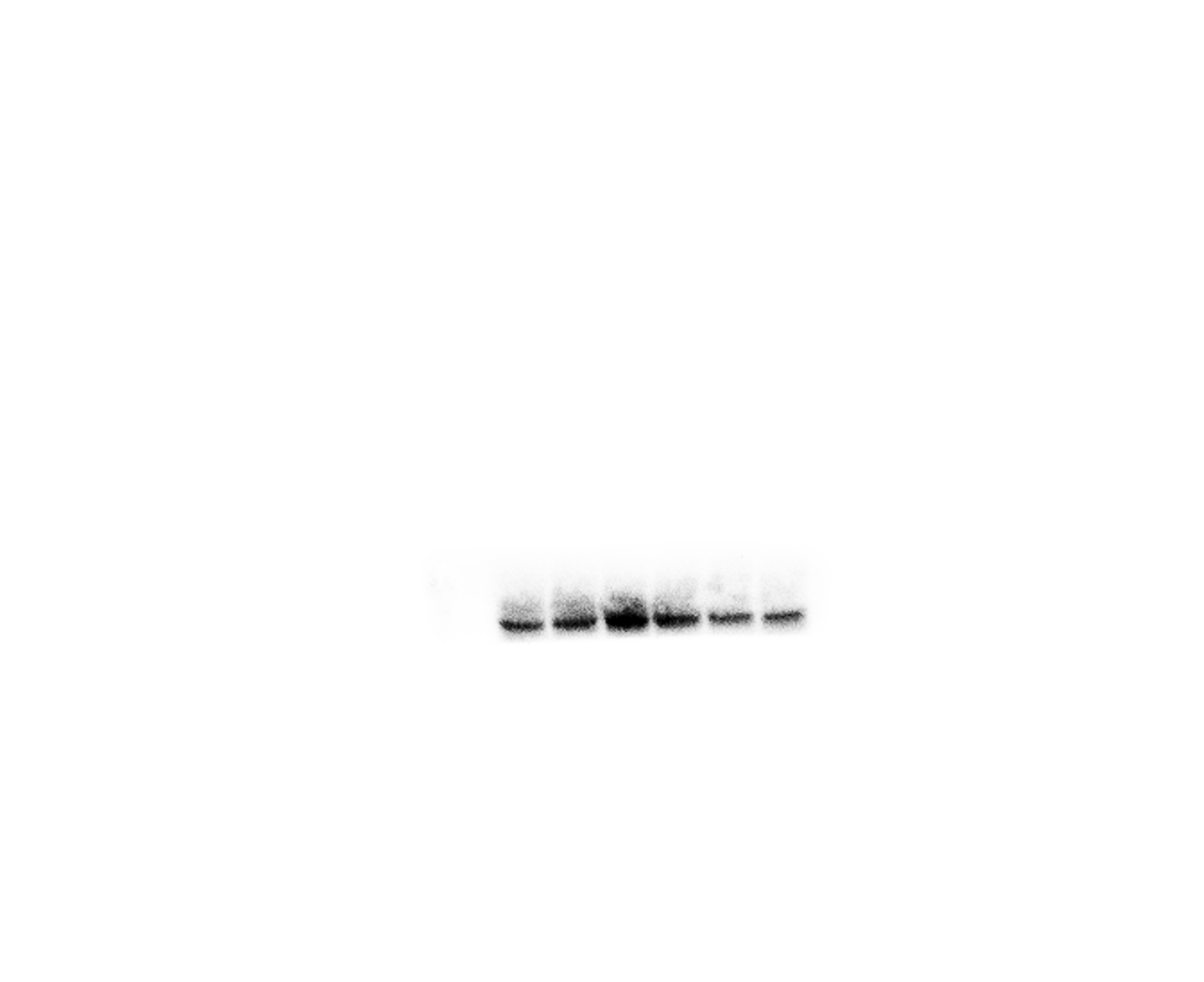

Supplement: Data Sheet 1 — The original images of western blot. The strips from left to right are control group, MA alone group, LPS group and LPS+MA (40, 20, 10 mg/kg). [file DataSheet_1.zip › Original image/p-ikb/p-ikb-1.tif]

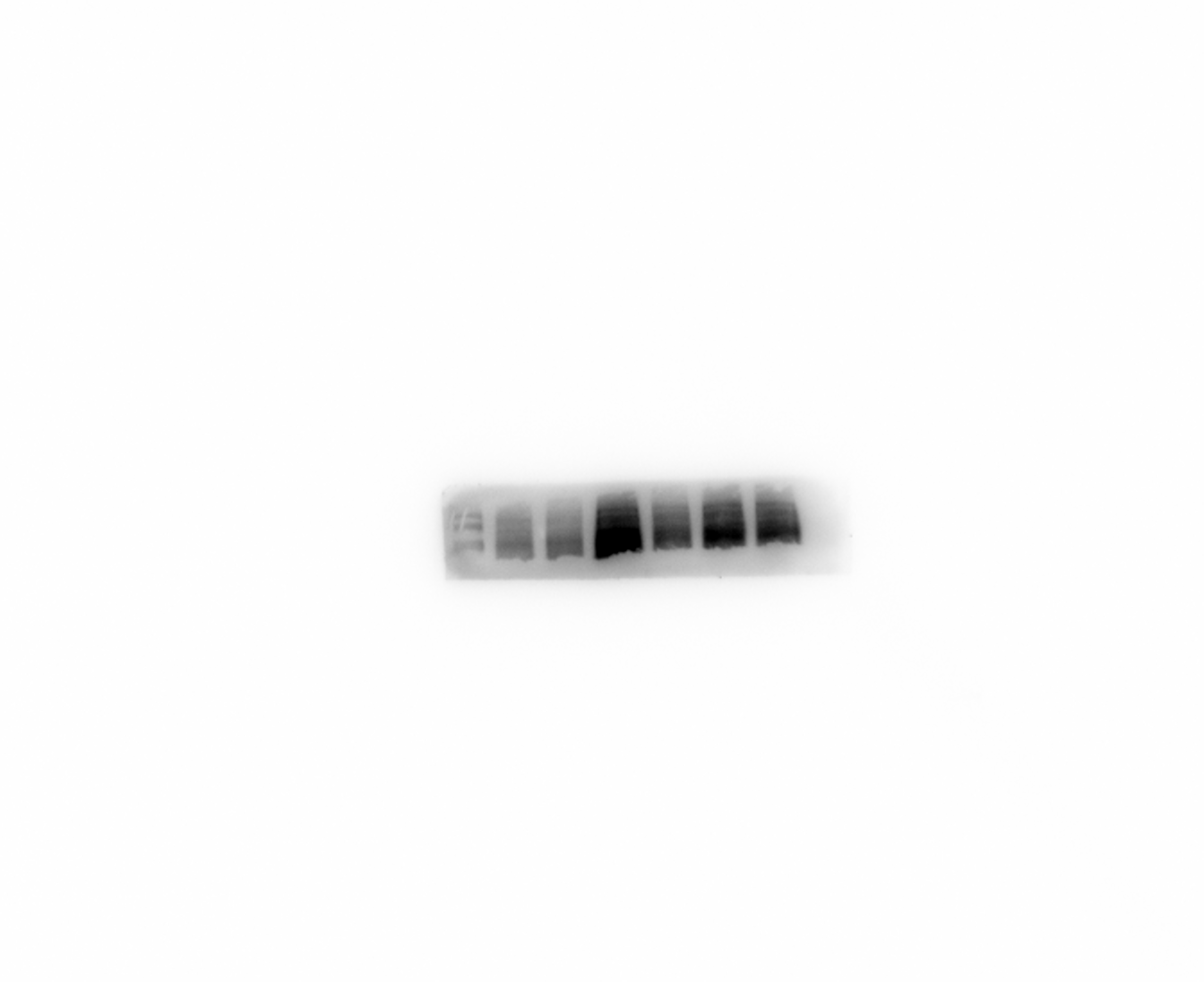

Supplement: Data Sheet 1 — The original images of western blot. The strips from left to right are control group, MA alone group, LPS group and LPS+MA (40, 20, 10 mg/kg). [file DataSheet_1.zip › Original image/p-ikb/p-ikb-2.Tif]

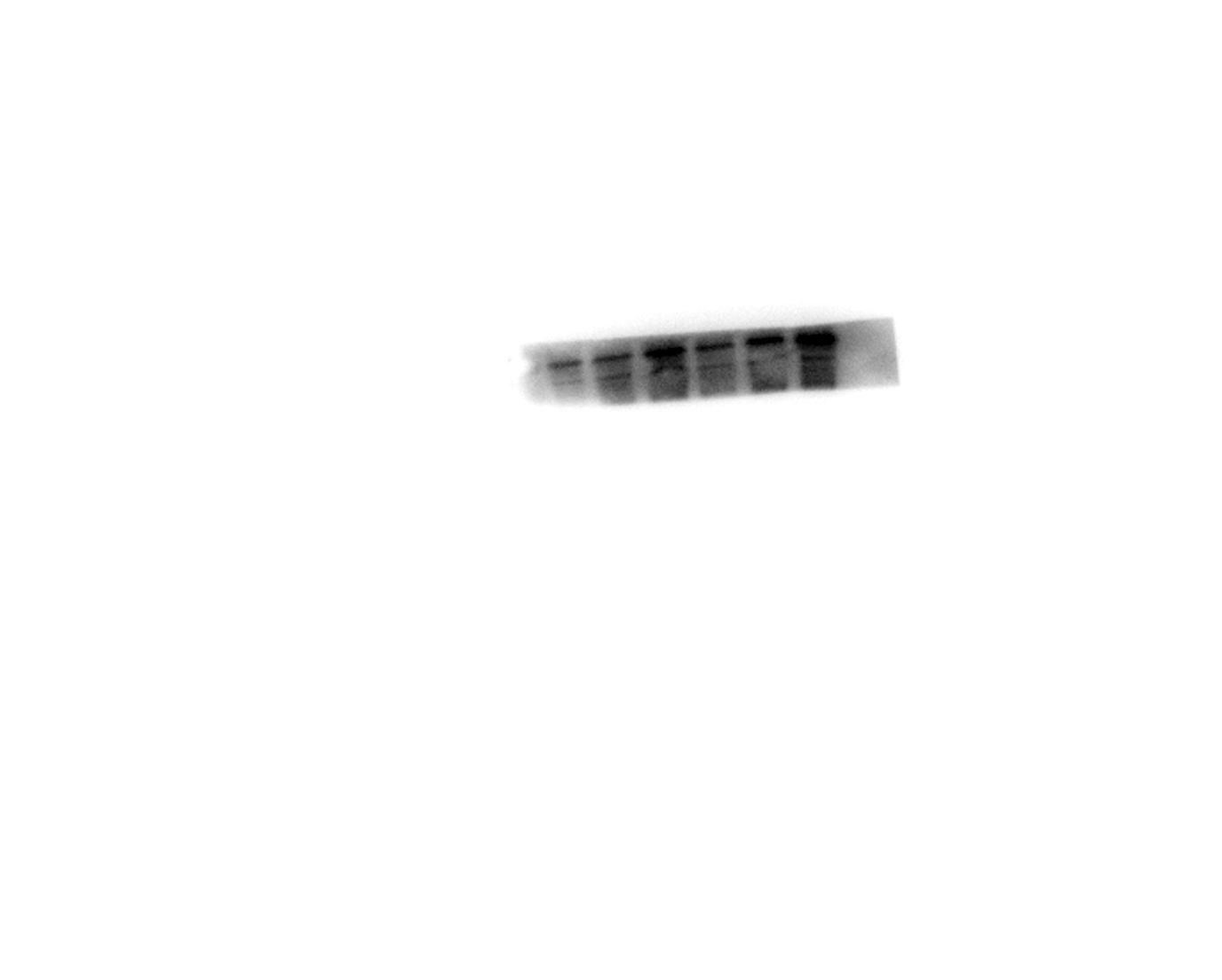

Supplement: Data Sheet 1 — The original images of western blot. The strips from left to right are control group, MA alone group, LPS group and LPS+MA (40, 20, 10 mg/kg). [file DataSheet_1.zip › Original image/p-ikb/p-ikb-3.tif]

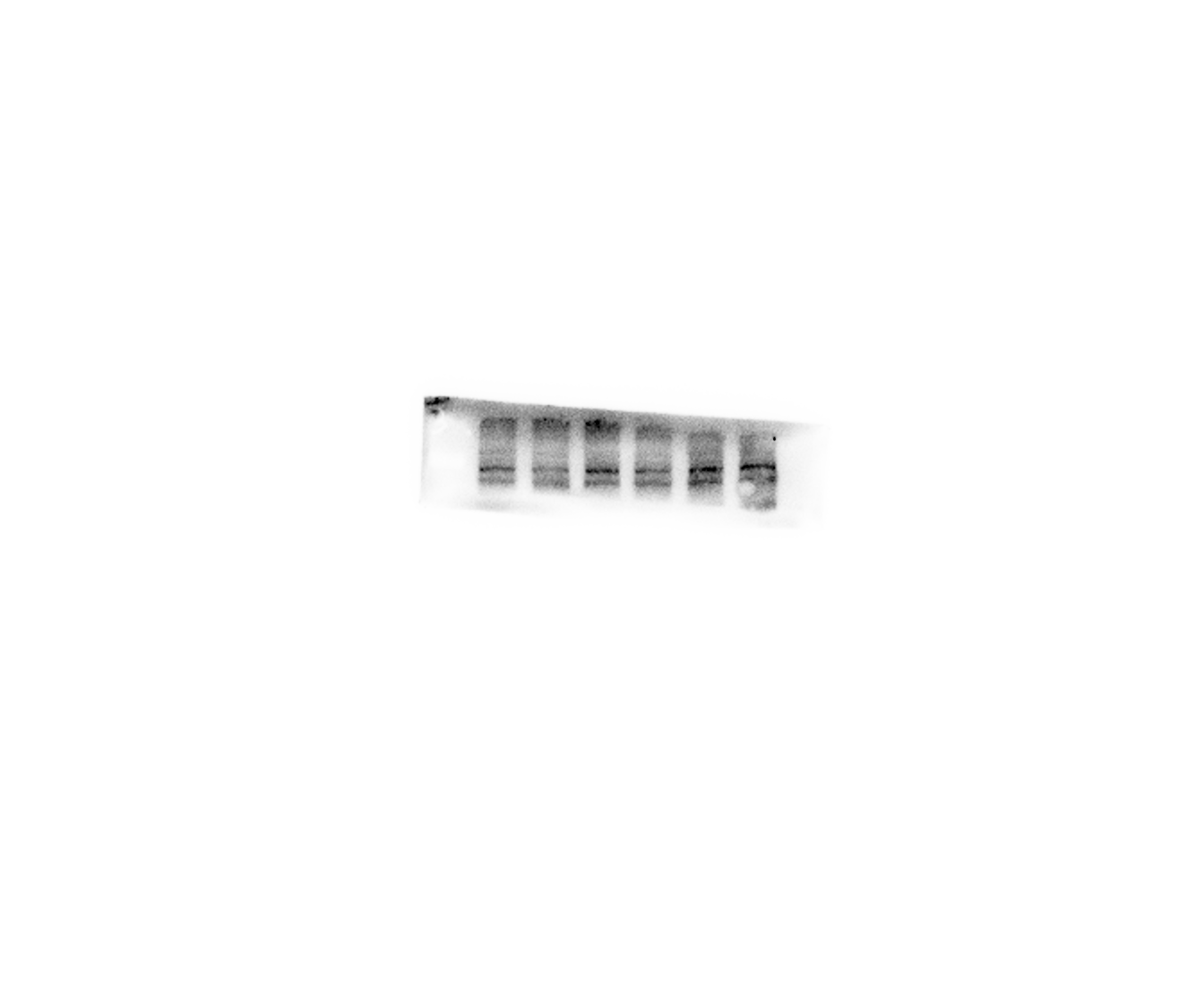

Supplement: Data Sheet 1 — The original images of western blot. The strips from left to right are control group, MA alone group, LPS group and LPS+MA (40, 20, 10 mg/kg). [file DataSheet_1.zip › Original image/p65/p65-1.tif]

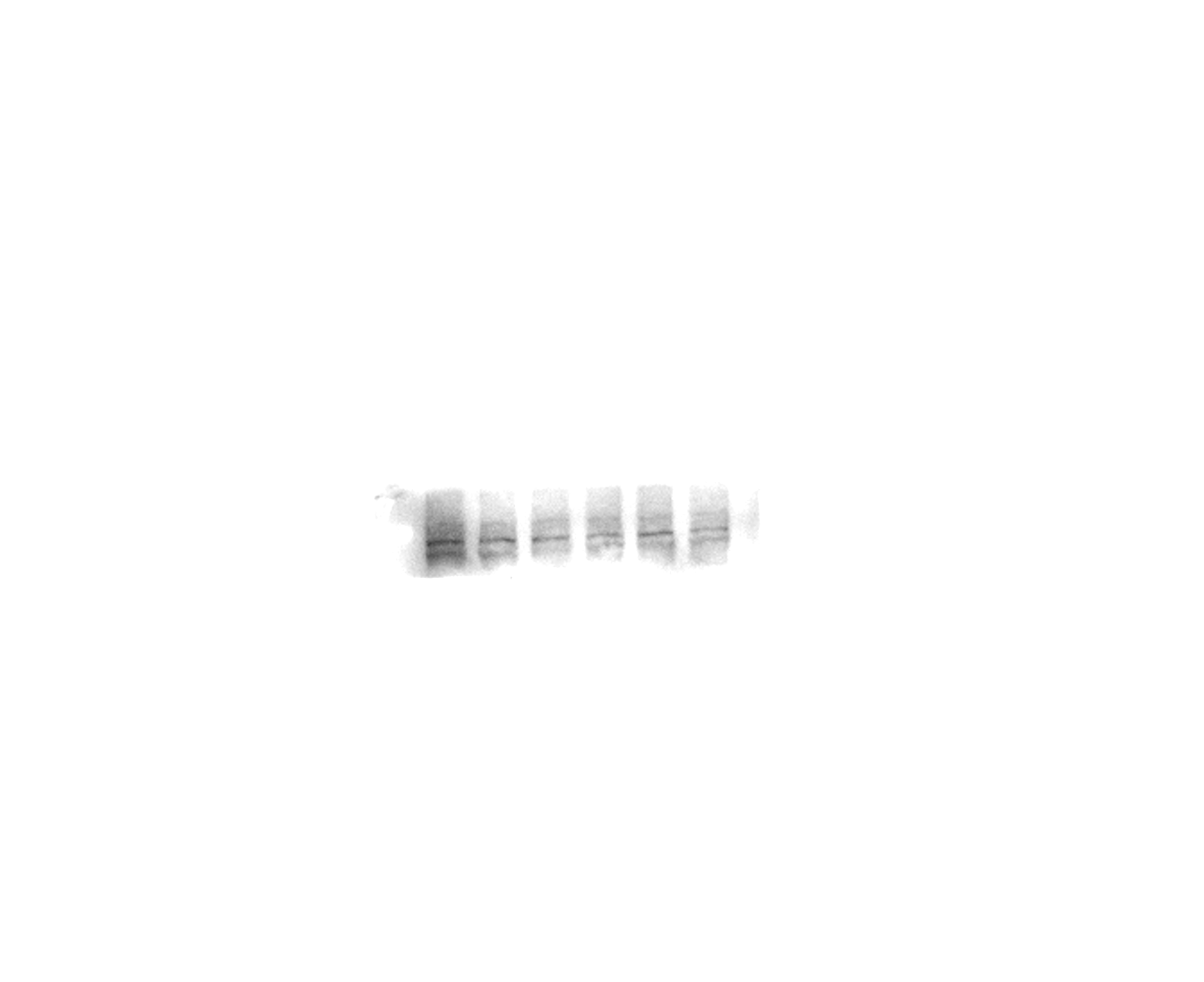

Supplement: Data Sheet 1 — The original images of western blot. The strips from left to right are control group, MA alone group, LPS group and LPS+MA (40, 20, 10 mg/kg). [file DataSheet_1.zip › Original image/p65/p65-2.Tif]

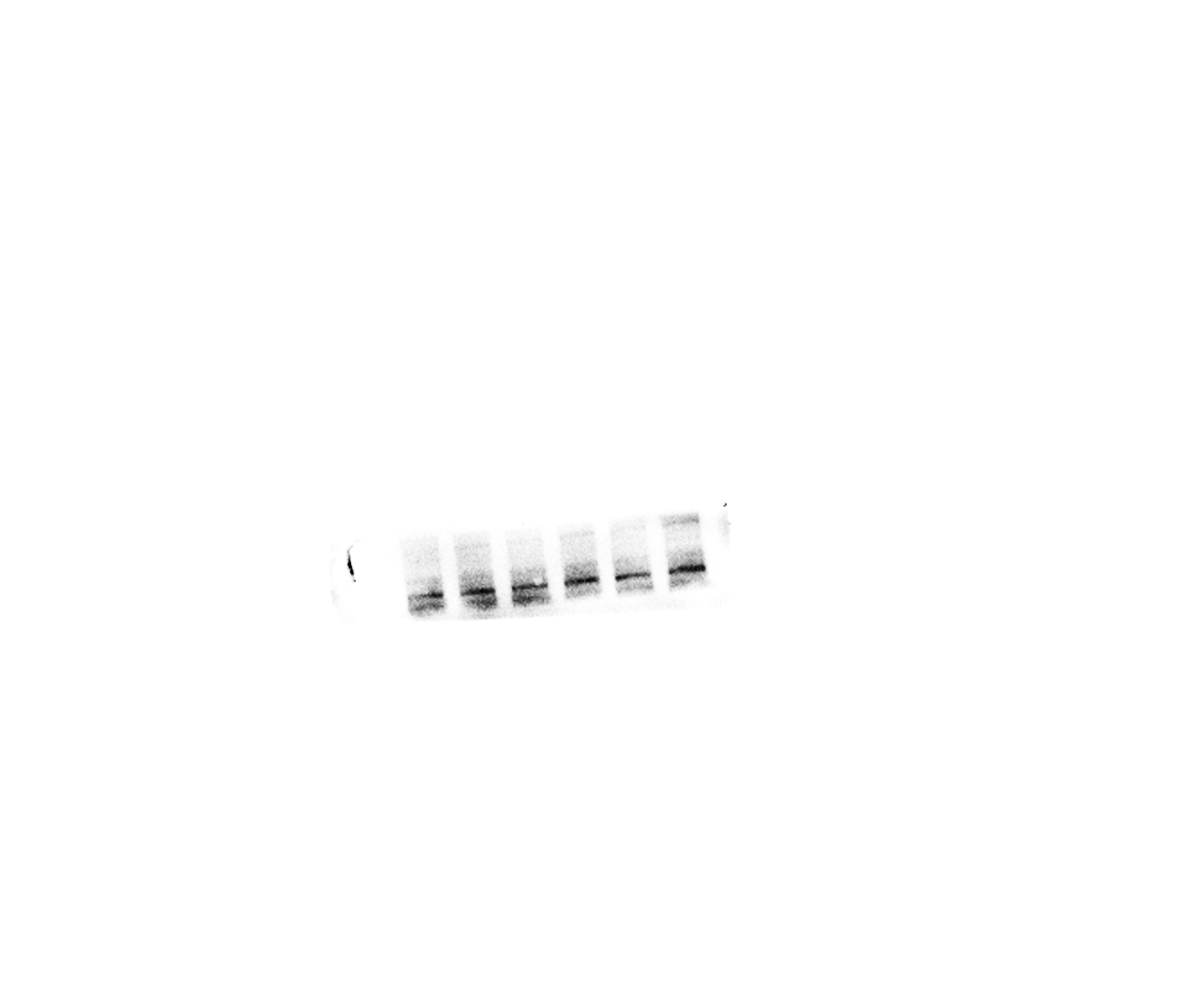

Supplement: Data Sheet 1 — The original images of western blot. The strips from left to right are control group, MA alone group, LPS group and LPS+MA (40, 20, 10 mg/kg). [file DataSheet_1.zip › Original image/p65/p65-3.tif]

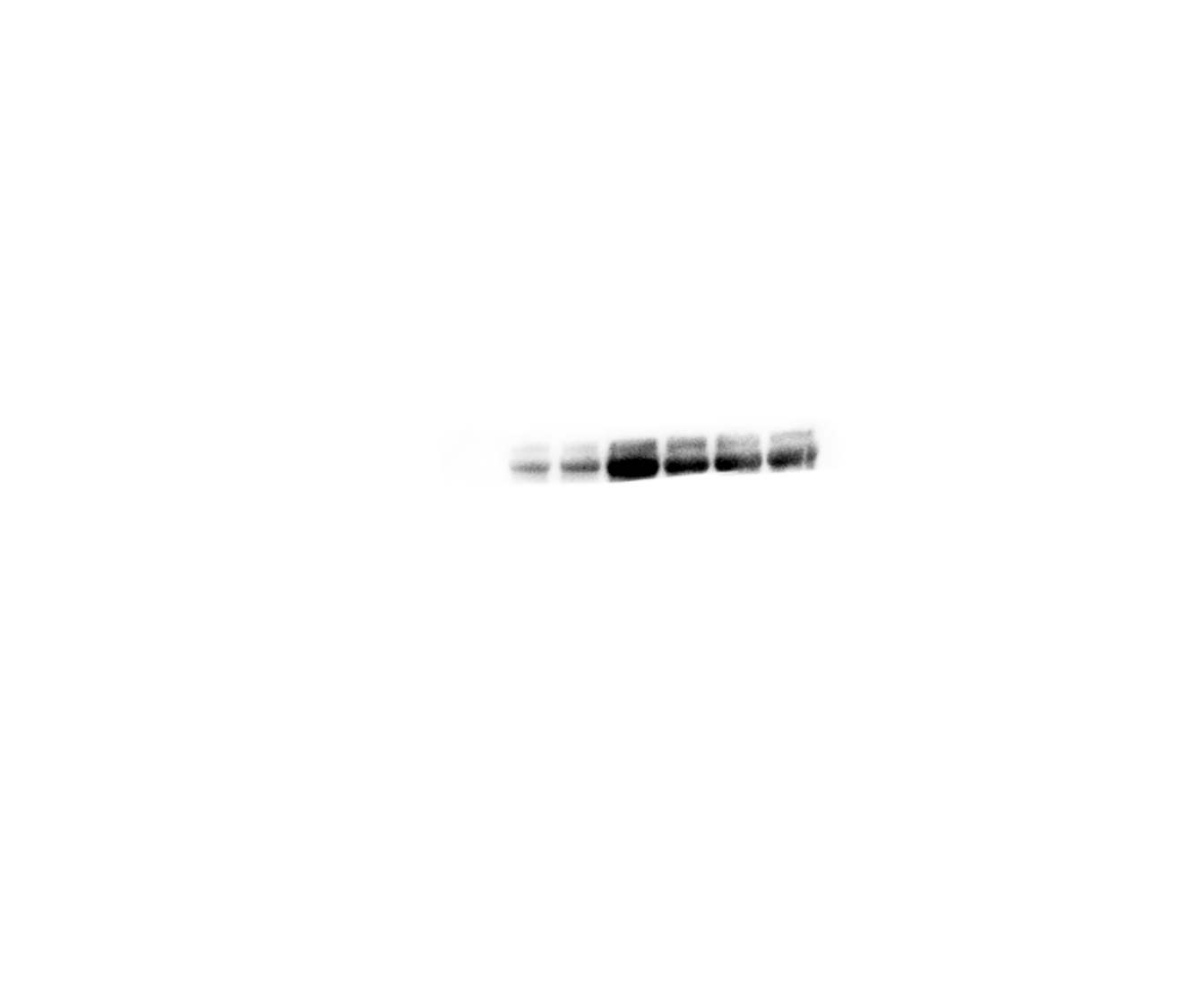

Supplement: Data Sheet 1 — The original images of western blot. The strips from left to right are control group, MA alone group, LPS group and LPS+MA (40, 20, 10 mg/kg). [file DataSheet_1.zip › Original image/pp65/pp65-2.tif]

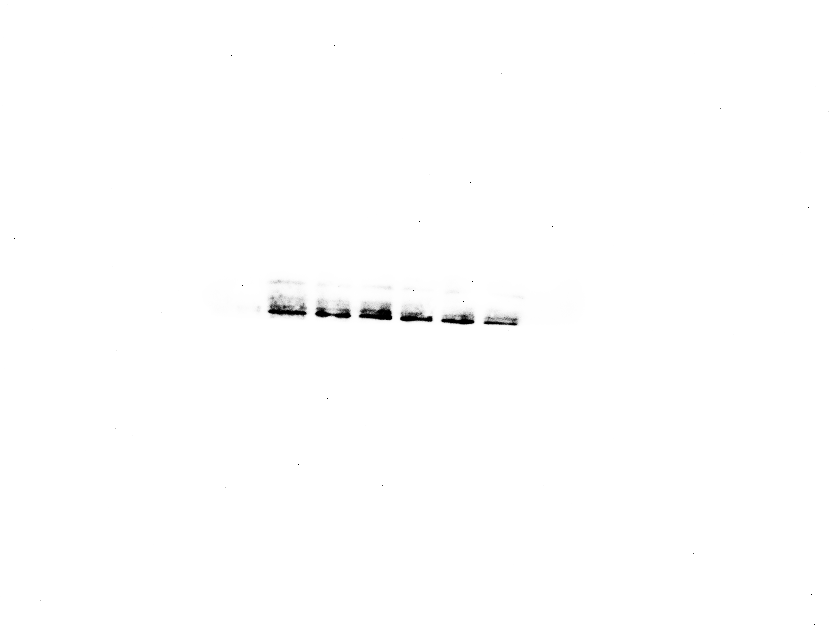

Supplement: Data Sheet 1 — The original images of western blot. The strips from left to right are control group, MA alone group, LPS group and LPS+MA (40, 20, 10 mg/kg). [file DataSheet_1.zip › Original image/pp65/pp65-3.tif]
